# Safety, Tolerability, and Pharmacokinetics of a Novel Mitochondrial Modulator, TRC150094, in Overweight and Obese Subjects: A Randomized Phase-I Clinical Trial

**DOI:** 10.3389/fphar.2021.729424

**Published:** 2021-09-17

**Authors:** Deepa Joshi, Prashant Jamadarkhana, Suchit Kumbhare, Amarinder Singh, Jignesh Kotecha, Deepak Bunger, Ajay Shiwalkar, Anookh Mohanan, Chaitanya Dutt

**Affiliations:** Torrent Pharmaceuticals Ltd., Ahmedabad, India

**Keywords:** TRC150094, insulin resistance, type 2 diabetes mellitus, hypertension, dyslipidemia, cardiometabolic based chronic disease, pharmacokinetics, mitochondrial modulator

## Abstract

TRC150094, a novel mitochondrial modulator, can restore metabolic flexibility by improving insulin resistance in preclinical studies. This study primarily aims to evaluate the safety, tolerability, and pharmacokinetics (PK) of oral TRC150094 after conducting two double-blind, randomized, Phase-I studies, single ascending dose (SAD) and multiple ascending dose (MAD), with *n* = 46, in overweight/obese adult and elderly subjects. In addition, the effect of TRC150094 on pharmacodynamic (PD) efficacy markers was evaluated. PK assessments, including maximum concentration (C_max_), area under the plasma concentration (AUC), time to C_max_ (T_max_), and elimination half-life (t½), were assessed at pre-specified time points. PD assessments included apolipoprotein B (ApoB), triglycerides, hepatic fat by magnetic resonance spectroscopy (MRS) and cardiopulmonary exercise testing (CPET) parameters. TRC150094 was rapidly absorbed, and the AUC of TRC150094 increased in a dose-dependent manner across all doses in non-elderly and elderly cohorts. C_max_ was more than the dose-proportional for all doses in all cohorts. T_max_ ranged from 0.25 to 4 h, and t½ ranged from 15 to 18 h, making TRC150094 suitable for once-daily dosing. Food did not interfere with the overall absorption of the drug. The metabolites of TRC150094 were glucuronide and sulfate conjugates, and 20% of the drug was excreted unchanged in the urine. TRC150094 at 50 mg showed an improving trend in triglycerides. A significant reduction in Apo B was observed after 50 mg dose (−2.34 vs. 13.24%, *p* = 0.008), which was, however, not the case after 150 mg (8.78 vs. 13.24%, *p* = 0.1221). Other parameters such as hepatic fat and insulin sensitivity indices (HOMA-IR, MATSUDA Index derived from OGTT) showed an improving trend for the dose of 50 mg. In terms of safety, all the AEs reported were mild to moderate in severity. None of the adverse events was considered definitely or probably related to treatment, and there were no abnormal laboratory findings. In conclusion, the PK of TRC150094 was linear with no clinically significant food effect. TRC150094 and its metabolites suggest a lesser likelihood of drug-drug interactions. Overall, TRC150094 ensured safety and exhibited suitability for all subjects.

**Clinical Trial Registration:** EUDRA CT: 2009-014941-10 (SAD) and CTR-India registration: CTRI/2009/091/000601 (MAD)

## Introduction

Type 2 diabetes mellitus (T2DM) is a chronic disease that presents a growing problem globally and that has reached alarming levels of severity (Saeedi et al., 2019). T2DM is a prevalent comorbidity of other chronic diseases, such as hypertension and dyslipidemia. The mortality risk due to cardiovascular (CV) events is higher when T2DM and hypertension co-exist (Sowers et al., 2001) and surges with the addition of dyslipidemia (Adler et al., 2000; Sowers et al., 2001). The development of T2DM, hypertension, and dyslipidemia can be attributed to diminished insulin sensitivity, i.e., insulin resistance (Sowers 2004).

Recently, this disease spectrum has been reclassified as dysglycemia-based chronic disease and cardiometabolic-based chronic disease (CMBCD) (Mechanick et al., 2020). The CMBCD model positions insulin resistance, prediabetes, T2DM, and related complications along a continuous spectrum. Further evidence suggests that the phenomenon of derangement of metabolic flexibility or metabolic inflexibility is a common thread linking insulin resistance to CMBCD, including dysglycemia, hypertension, and/or dyslipidemia, progressing to micro-and macro-vascular complications, such as atherosclerotic vascular disease (Sergi et al., 2019; Kalra et al., 2021).

Traditional glucocentric approaches have failed to control disease progression across the spectrum, and only half of the individuals could achieve accepted treatment goals. In addition, an aggressive strategy, such as intensive glycemic control, has failed to reduce mortality and cardiovascular outcomes in those with T2DM. A previous study has reported the possible contribution of mitochondrial dysfunction to the pathological state (Camara et al., 2010). Therefore, targeting mitochondria may represent a valuable therapeutic tool for improving insulin resistance and reducing the cardiovascular risk in subjects with T2DM, dyslipidemia, and hypertension (Kalra et al., 2021).

TRC150094, a novel mitochondrial modulator, is developed, which demonstrates the ability to restore metabolic inflexibility caused by mitochondrial dysfunction. In preclinical studies, systemic administration of TRC150094 to high fat diet rats led to an increased activity of electron transport chain complex II and V and increased mitochondrial fatty acid import and oxidation, leading to increased energy expenditure (Cioffi et al., 2010; Silvestri et al., 2012). The effects mimic functionally, and stimulation of mitochondrial bioenergetic mechanisms are observed with di-iodothyroinine (Goglia 2005). TRC150094 administered to obese Zucker fatty and spontaneously hypertensive (ZSF1) rats attenuated the progression of insulin resistance, dysglycemia, atherogenic dyslipidemia, and hypertension (Zambad et al., 2011). Moreover, extensive safety evaluation, including long-term toxicity studies on rodent and non-rodent species in which target-related effects on thyroid axis, cardiac, bone, and cartilage were specifically monitored, did not reveal study drug-related changes in the biomarkers or histology. In addition, the respiratory, central nervous system, and cardiovascular safety evaluation in sensitive animal models (either rodent or dog) confirmed a wide safety margin. An improving trend in triglyceride levels in overweight/obese subjects has been reported (van der Valk et al., 2014). This paper describes the results of two Phase-1 studies, single ascending dose (SAD) and multiple ascending dose (MAD), conducted for evaluating the safety, tolerability, pharmacokinetics (PK), and exploratory pharmacodynamics (PD) parameters of TRC150094 ranging from 5 to 400 mg in overweight/obese subjects.

## Methods

The SAD study was conducted at Biotrial, Rennes, France (registered as EUDRA CT: 2009–014941–10). The MAD study was conducted at Lambda Therapeutic Research Ltd., Ahmedabad, India [registered in the Clinical Trial Registry–India (Identifier number: CTRI/2009/091/000601)]. The study protocols were reviewed and approved by the respective institutional review boards and concerned regulatory authorities before the conduct of the study.

The SAD study was approved by Comite de Protection des Personnes Ile de France VII (Hôpital de Bicêtre - 78, rue du Général Leclerc - 94270 Le Kremlin Bicêtre), and the MAD study was approved IEC-Aditya, Ahmedabad, India. Both clinical studies were conducted in accordance with Good Clinical Practice guidelines and the Declaration of Helsinki (WMA Association, n.d.).

### Single Ascending Dose Study

#### Subjects

This study was conducted in two parts: Part A in adults (18–65 years) and elderly (>65 years) overweight/obese male and female and Part B (food effect study) in six healthy adult subjects (18–65 years). The key inclusion criteria for Part A were subjects with body mass index (BMI) of 25–40 kg/m^2^, non-smokers or smoker subjects smoking ≤10 cigarettes/day but willing to stop smoking during the study duration, and subjects without significant comorbidities. In Part B, subjects with BMI of 18–25 kg/m^2^ were enrolled, with the remaining inclusion criteria similar to Part A. The key exclusion criteria were Alanine transaminase (ALT) or Aspartate transaminase (AST) ≥ 2 times the upper limit of normal, with eGFR <40 ml/min/1.73 m^2^ (as evaluated by Modification of Diet in Renal disease (MDRD) method).

#### Study Design

This study was a double-blind, randomized, placebo-controlled, sequential, single-dose, dose-escalation study to evaluate the safety, tolerability, and PK of TRC150094 administered orally to overweight/obese adults and elderly subjects. TRC150094 was administered as a tablet in strengths of 2.5, 12.5, 50, and 200 mg, which were manufactured as per Good Manufacturing Practice (GMP). Tablets were bulk supplied in high density polyethylene bottles (30’s count) containing desiccant and cotton, with an appropriate label as per GMP.

The subjects in Part A of the study were assigned to four cohorts. Adult subjects in cohorts 1–3 (*n* = 8; active:placebo = 6:2) were randomized to two incremental dose levels of 5, 25, 50, 100, 200, and 400 mg or placebo in an intercalated crossover design (Table 1). Elderly subjects (*n* = 16; active:placebo = 12:4) in cohort four received a single dose of 50 and 150 mg or placebo in a crossover design. A washout period of at least 7 days was maintained between two dose levels.

**TABLE 1:** Dose escalation in the single ascending dose study.

Subjects (*n* = 40)	Dose administered							
Cohort 1 (*n* = 8, 6:2)	Dose 1			Dose 4				
	5 mg			100 mg								
Cohort 2 (*n* = 8, 6:2)		Dose 2			Dose 5			
		25 mg			200 mg						
Cohort 3 (*n* = 8, 6:2)			Dose 3			Dose 6		
			50 mg			400 mg				
Cohort 4 Elderly cohort (*n* = 16, 12:4)							Dose A	Dose B
							50 mg	150 mg

If a dosing regimen (beginning with the lowest dose) was found to be safe and well-tolerated by the Dose Review Committee (DRC), then the succeeding panel of eight subjects received the next higher dose of TRC150094 or placebo. The DRC was blinded when assessing the PK, safety, and tolerability data following each dosing occasion. The committee also confirmed dose modification and the highest dose level to be achieved. Considering no observed adverse effect levels (NOAEL) in rodents and dogs, the proposed starting dose level for SAD was 5 mg, which was administered to subjects with an approximate weight of 90 kg (1/260th and 1/750th of the NOAEL in rats and dogs, respectively). The maximum permitted dose level of TRC150094 of 400 mg (1/3rd and 1/9th of the NOAEL in rats and dogs, respectively) was considered appropriate by the DRC.

**FIGURE 1:**
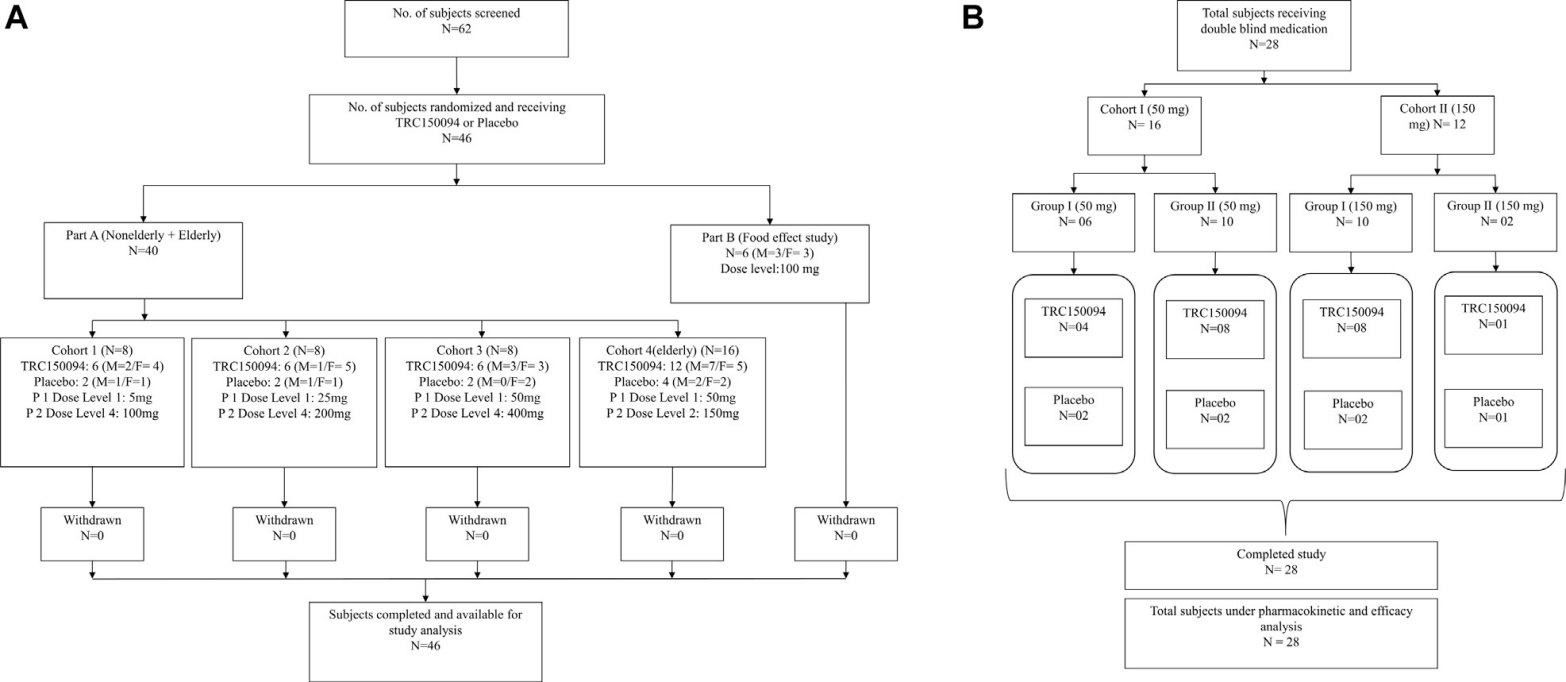
Study design and subject disposition in **(A)** single ascending dose study and **(B)** multiple ascending dose study.

Part B was an open-label food effect study in which six subjects were randomized to receive 100 mg of TRC150094 in fasting and fed conditions in a 2-period crossover fashion. Figure 1A presents the study design and dosing. Subjects fasted for 10 h and received a standard meal of 500 kcal 30 min prior to dosing and consumed within 30 min in the fed study. No food was permitted prior to and for 4 h post-dose in the fasting study. Subsequently, standardized meals (400–500 Kcal) were provided at 4, 10, and 12 h post dose to both treatment groups.

The duration of study for each subject was approximately 37 days, including screening (14 days), treatment period (total of 6 days in two sequential study periods: period I and period II of 3 days each), washout period (at least 7 days), and follow-up (8–10 days after last study treatment administration). Blood samples (7 ml) were collected at 0 (pre-dose), 0.16, 0.33, 0.5, 0.75, 1, 1.25, 1.5, 2, 3, 4, 6, 8, 12, 16, 24, 36, and 48 h post-dose for all dose levels to measure the concentrations of TRC150094. Urine samples were collected in the intervals of pre-dose (bladder was emptied before dosing), 0–4, 4–8, 8–12, 12–24, 24–36, and at 36–48 h post-dose after dose 4 (100 mg) in adult and dose B (150 mg) in the elderly cohort in Part A.

### Multiple Ascending Dose Study

#### Subjects

Overweight/obese male and female subjects (18–65 years) with BMI 27–35 kg/m^2^; waist circumference ≥90 and ≥80 cm for men and women, respectively; nonalcoholic fatty liver grade 1–3 [based on Magnetic Resonance Spectroscopy (MRS)]; fasting serum insulin ≥10 mU/ml; fasting plasma glucose >100 mg/dl; systolic blood pressure ≥130 mmHg; and/or diastolic blood pressure ≥85 mmHg were enrolled. Furthermore, subjects on antihypertensive medications and fasting serum triglyceride (TG) ≥150 mg/dl, as well as non-smokers (or subjects smoking ≤10 cigarettes/day but willing to stop smoking during the study duration), were enrolled. Female subjects, with/without childbearing potential, using a highly effective method of contraception, except for oral contraceptive pills, at least 15 days prior to enrolment till 10 days after the last dose were included. The exclusion criteria were blood pressure ≥170/110 mmHg and ALT or AST ≥3 * ULN, and eGFR <60 ml/min/1.73 m^2^. All subjects provided written informed consent to participate in the study.

#### Study Design

The study was a double-blind, single-center, placebo-controlled, multiple-dose, dose-ascending study to assess the safety, tolerability, and PK and to explore the early efficacy markers after multiple oral doses of TRC150094 in overweight/obese male and female subjects with nontraditional CV risk factors.

The subjects were randomly assigned to TRC150094 (50 mg or 150 mg) or placebo in a ratio of 3:1 for a treatment duration of 28 days. Each subject attended the clinical facility for a screening visit, one study period, and a post-study follow-up visit (Day 36–38). Subjects were admitted and housed in the clinical facility at least 2 days before administration of the first dose and continued to remain in the clinical facility for at least 48 h (Day 30) after receiving the last dose of the investigational medicinal product. All the subjects remained in the clinical facility for 31 consecutive nights. Subjects returned to the clinical facility for a follow-up visit 8–10 days after their last dose. Dosing was performed in the morning for 28 days (OD) in a fasting state of at least 8 h. For each subject, dosing time throughout the study period was based on the dosing time of Day 1. Blood samples (8 ml) were taken for PK analysis at Day 1: Pre-dose, 0.25, 0.5, 0.75, 1, 1.25, 1.5, 2, 3, 4, 6, 12, 18, and 24 h post-dose; Day 3–8, 15, 22: Pre-dose; and Day 28: Pre-dose, 0.25, 0.5, 0.75, 1, 1.25, 1.5, 2, 3, 4, 6, 12, 18, 24, 48 h, ambulatory 72 h, and 96 h post-dose. The urine samples were collected for analysis on Day 1: pre-dose collection (bladder was emptied before dosing), post-dose 0–4, 4–8, 8–12, 12–24 h; Day 28: pre-dose collection, 0–4, 4–8, 8–12, 12–24, 24–36, and post dose, 36–48 h (relative to the dose on Day 28). Figure 1B depicts a schematic presentation of the study design.

### Safety Evaluations

Safety evaluations were similar in the two studies and were based on the medical review of adverse event (AE) reports. Clinical examination, vital signs, chest X-ray, electrocardiogram (12 lead ECG), clinical laboratory parameters (biochemistry and serum electrolytes, hematology, lipid parameters, urinalysis, and serology), and specific renal biomarkers (urinary alpha-glutathione S transferase for proximal tubular damage) were considered. Fecal occult blood test (FOBT) based on preclinical toxicity findings at highest dose and AE monitoring were performed as part of the safety and tolerability evaluations in both studies. Severity was graded as mild, moderate, and severe along the lines of Common Terminology Criteria for Adverse Events.

### Pharmacokinetics Assessments

The PK parameters, namely the maximum concentration (C_max_), area under the plasma concentration-time curve (AUC) from time zero to the last measurable concentration (AUC _o-last_), time to C_max_ (T_max_), AUC from time zero to infinity (AUC _0-∞_), terminal rate constant (λz), and elimination half-life (t½), were analyzed for all doses in both studies. Concentrations of TRC150094 (50 and 150 mg doses) and its metabolites (150 mg dose), which are conjugated metabolite-1 (M1; acyl glucuronide), metabolite-2 (M2; hydroxy glucuronide), and metabolite-3 (M3; sulfate), were measured in the MAD study and were determined using sensitive liquid chromatography-tandem mass spectrometry (LC-MS/MS) in plasma and urine at the bioanalytical laboratory, Torrent Research Centre, Gandhinagar, India. All the assays in plasma and urine analyses were validated according to the United States Food and Drug Administration (USFDA) guidance for bioanalysis (U.S. Food and Drug Administration, 2018). PK parameters were derived for plasma and urine data of TRC150094 in 50 and 150 mg doses and its metabolites M1, M2, and M3 for 150 mg dose.

TRC150094 and its metabolites were determined in plasma and urine via solid phase extraction (SPE; Waters Corporation, Milford, MA, United States), followed by reversed-phase chromatography using beta basic C8 column (Thermo Fisher Scientific, San Jose, CA, United States) and Zorbax SB-C18 column (Agilent Technologies Inc., Santa Clara, CA). In addition, Shimadzu HPLC, comprising SIL-HTc autosampler, LC-20AD Solvent Delivery Module, CTU-20A Column Oven, and DGU-20A5 Degasser (Shimadzu Corporation, Kyoto, Japan), coupled with an API4000 triple quadrupole mass spectrometer (AB SCIEX, Framingham, MA) or TSQ Vantage triple quadrupole mass spectrometer (Thermo Fisher Scientific, San Jose, CA, United States), was used. Detailed information on analytical methods are presented in the Supplementary Material.

The quantification ranges for TRC150094 were 0.5–200 (5/25 mg) and 5–2000 ng/ml (50/100/200/400 mg) for plasma, and 0.1–40 μg/ml for urine (all doses). For metabolites M1, M2, and M3, the quantification ranges were 5–2000 ng/ml, 5–1,000 ng/ml, and –1,500 ng/ml, respectively, for plasma and 0.1–40 μg/ml, 50–5,000 ng/ml, and 10–1,000 ng/ml, respectively, for urine.

### Pharmacodynamics Marker Assessment

In the MAD study, non-traditional cardiovascular risk factors, including hepatic fat, ApoB, and insulin sensitivity, were evaluated as early efficacy PD markers after TRC150094 administration.

#### Insulin Sensitivity

The insulin sensitivity was assessed by oral glucose tolerance test (OGTT). A 75 gm oral glucose load was administered, and the blood samples were collected at 0, 30, 60, 90, 120, 180 min for glucose and insulin measurements. HOMA-IR and MATSUDA Index were derived using the following formulas:

○ HOMA-IR (mg/dl × μU/mL) was calculated as:
= fasting Glucose (mg/dl) × fasting Insulin (μU/mL)/405
○ MATSUDA Insulin Sensitivity Index =
10000÷√[(FPG (mmoL/L) × FPI (mU/L) × (mean OGTT glucose concentration) × (mean OGTT insulin concentration)]
(where FPG = fasting plasma glucose, FPI = fasting plasma insulin)

#### Hepatic Fat by MRS

Hepatic fat was measured at screening, baseline, and at the end of the study. MRS was performed by a 3.0 T MRI using SENSE XL Torso coil (Philips) and single-voxel MRS, with the torso coil as the transmitter and phased surface coils as the receiver. MRS measurements were acquired during breath-hold using single-voxel stimulated acquisition mode (TE/TR 20/3.000 ms, six acquisitions) at a voxel size of 27 mm^3^. Data analysis and interpretation were conducted using jMRUI software version 3. x (proprietary software package for advanced analysis of MRS data) at the Academic Medical Center, Amsterdam, the Netherlands.

#### Oxygen Uptake Efficiency Slope and V_O2_ Max

These were measured by cardiopulmonary exercise testing (CPET) using a metabolic cart (Innocor). The CPET parameter oxygen uptake efficiency slope (OUES) was obtained at screening, baseline, and at the end of the study from the data obtained during the properly identified exercise phase. OUES was calculated as the slope derived from the equation: VO_2_ = a log10 V_E_ + b, where a represents OUES, and V_E_ represents minute ventilation. The VO_2_ and V_E_ data obtained from the exercise phase was used for this calculation. Peak VO_2_ (VO_2__max_) was estimated as maximal V_O2_ observed during the end of the exercise phase and the first 30 s of the recovery phase. Other markers measured were serum TG, apolipoprotein B (Apo B).

### Statistical Analysis

For TRC150094 and its three metabolites, the WinNonlin® Version 5.0.1 and 5.3 (Certara L.P., Princeton, NJ, United States) was used to estimate PK parameters in plasma (C_max_, T_max_, AUC_0-t_, AUC_0-24_, AUC_0-∞_, K_el_, and t_1/2_) and urine (AURC_0-24_ and AURC_0-48_, with 24 and 48 h renal clearance, respectively) were estimated, and to prepare the dataset. SAS® Version 9.2 (SAS Institute Inc., United States) was used to generate the randomization code and perform statistical analysis. A formal sample size calculation was not conducted for the SAD and MAD studies.

Descriptive statistics were calculated for plasma concentration and PK parameters of TRC150094 and its three phase II metabolites. The effect of food on PK was evaluated based on logarithmically transformed PK parameters (C_max_ and AUC_0-∞_) using an analysis of variance (ANOVA) model.

Dose proportionality of PK parameter estimates was evaluated based on the derived PK parameters, C_max_ and AUC_0-24_, separately for days 1 and 28, along with AUC_0-∞_ for Day 28, in the MAD study. The PK parameters and doses were logarithmically transformed before conducting statistical analysis using the power model (Gough et al., 1995). A linear mixed-effects model comprising dose, sex, and dose*sex as a fixed effect, and subject as a random effect, was fitted to the data. The dose proportionality was assessed using the estimated slope parameter of the linear model and 90% confidence interval (CI) derived on the dose at each day assessed. Non-parametric Wilcoxon Sign rank test was performed to compare T_max_ between days 1 and 28 for each dose of TRC150094 and its metabolites.

The accumulation of TRC150094 was evaluated based on the AUC_0-12_ and AUC_0-24_ after their logarithmic transformation. The analysis was performed using an ANOVA mixed-effects model, comprising fixed effects for sex, day, dose, and dose-by-day interaction, with subject-within-dose as a random effect. A 95% CI for mean between-day difference (Day 28 AUC minus Day 1 AUC) was calculated by dose using the ANOVA model for C_max_, AUC_0-12_, and AUC_0-24_.

A 95% CI for least-square means (LSM) for days 1 and 28 was calculated for each dose using the ANOVA model, with fixed effects for log (dose), sex, log (dose)*sex for ln transformed C_max_, AUC_0-12_, AUC_0-24,_ and AUC_0-∞_. These CIs were exponentiated to obtain the confidence interval for each dose.

The time to achieve a steady-state plasma concentration of TRC150094 for each dose and metabolite(s) was evaluated in trough predose plasma concentration (C_PD_) collected during multiple-dose administrations at each dose level in the MAD study. The evaluation was performed after logarithmic transformation at each dosing level using a repeated measure ANOVA model.

For PD efficacy markers, % change observed after 28 days to baseline in each group for MATSUDA Index, HOMA-IR, OUES, Peak V_O2_, Hepatic Fat, Apo B, and Triglycerides were subjected to ANOVA with fixed effect as ‘dose’ adjusted for ‘Change in body weight’ (multiple comparisons using Dunnett test).

Changes observed at baseline and after 28 days in each group for MATSUDA Index, HOMA-IR, OUES, Peak V_O2_, Hepatic Fat, Apo B, and Triglycerides were subjected to paired *t*-test.

## Results

### Demographics and Disposition

A total of 46 subjects (part A: *N* = 40, part B: *N* = 6) enrolled and completed the SAD study. Out of 40 overweight/obese subjects in Part A study, 24 and 16 subjects were enrolled in the adult and elderly cohorts, respectively. The remaining six healthy subjects (3 males and females each) were enrolled in the food effect study (Part B) (Figures 1A,B). The baseline demographic characteristics and smoking status of the study subjects were comparable between the groups (Table 2).

**TABLE 2:** Demography and baseline characteristics.

Variable	Categories	SAD					MAD		
		Adult (Part A)		Elderly (Part A)		Food effect (Part B)			
		TRC150094	Placebo	TRC150094	Placebo	100 mg	TRC150094	TRC150094	Placebo
		5–400 mg	(*N* = 6)	50/150 mg	(*N* = 4)	(*N* = 6)	50 mg	150 mg	(*N* = 7)
		(*N* = 18)		(*N* = 12)			(*N* = 12)	(*N* = 9)	
**Gender, n (%)**	Female	12 (66.7)	4 (66.7)	5 (41.7)	2 (50.0)	3 (50.0)	3 (33.3)	4 (44.4)	2 (22.2)
	Male	6 (33.3)	2 (33.3)	7 (58.3)	2 (50.0)	3 (50.0)	9 (47.4)	5 (26.3)	5 (26.3)
**Race, n (%)**	Black/African	1 (5.6)	3 (50.0)	0	0	1 (16.7)	0	0	0
	Other (African/White) Mixed	1 (5.6)	0	0	0	1 (16.7)	0	0	0
	Other: Metis	0	0	1 (8.3)	0	0	0	0	0
	Other: West Indies	1 (5.6)	0	0	0	0	0	0	0
	White	15 (83.3)	3 (50.0)	11 (91.7)	4 (100.0)	4 (66.7)	0	0	0
	Indian	0	0	0	0	0	12 (100)	9 (100)	7 (100)
Age (years) *	All Subjects	46.1 (15.3)	42.3 (19.1)	70.4 (4.8)	69.5 (0.5)	50.0 (7.7)	39.7 (11.8)	46.0 (5.7)	45.1 (10.5)
Height (cm) *	All Subjects	167.9 (6.5)	165.3 (6.2)	164.8 (10.3)	161.5 (7.1)	165.7 (7.9)	164.9 (7.8)	161.2 (6.7)	161.3 (7.8)
Weight (kg) *	All Subjects	86.5 (15.0)	79.0 (8.2)	77.8 (12.0)	72.5 (4.4)	63.8 (10.0)	84.3 (10.0)	77.3 (6.7)	82.6 (12.3)
**BMI (kg/m** ^**2**^ ) *	All Subjects	30.6 (3.9)	28.9 (2.6)	28.5 (1.6)	27.8 (0.8)	23.2 (2.1)	30.9 (2.4)	29.8 (2.6)	31.6 (2.4)
**Smoking status, n (%)**	**Smoker**	2 (11.1)	2 (33.3)	2 (16.7)	0 (0.0)	-	0 (0.0)	0 (0.0)	1 (14.3)
	**Ex-smoker**	6 (33.3)	2 (33.3)	5 (41.7)	1 (25.0)	-	3 (25.0)	1 (11.1)	0 (0.0)

*N* = Number of subjects with non-missing values; n/% = Number/percentage of subjects with the given characteristics; * = Mean (SD).

Abbreviations: BMI, body mass index; SAD, single ascending dose; MAD, multiple ascending doses.

### Safety Findings

TRC150094 was found to be well-tolerated in single and multiple doses. There were no deaths, serious AEs (SAEs), or discontinuation due to AEs observed in the study. All the AEs reported were mild to moderate in severity, and none of the AEs were considered definitely or probably related to treatment. In both SAD and MAD studies, the treatment-emergent AEs reported included decreased appetite, back pain, myalgia, pain in extremity, dizziness, headache, pyrexia, and cough (Table 3). These were isolated incidences that were not dose-related. All subjects had eGFR above 60 ml/min/1.73 m^2^ at screening and remain unchanged at the end of the visit.

**TABLE 3:** Summary of adverse events.

Adverse event		SAD/Adult								SAD elderly (Part A)			MAD		
System organ class (SOC)	Preferred term (PT)	TRC150094 5-400 mg n (%)							Placebo	TRC150094		Placebo	TRC150094		Placebo (*N* = 7) n (%)
		5^a^ mg (*N* = 6)	25^b^ mg (*N* = 6)	50^c^ mg (*N* = 6)	100^a^ mg (*N* = 6)	200^b^ mg (*N* = 6)	400^c^ mg (*N* = 6)	All (*N* = 36)	(*N* = 12) n (%)	50 mg (*N* = 12)	150 mg (*N* = 12)	(*N* = 8) n (%)	50 mg (*N* = 12) n (%)	150 mg (*N* = 9) n (%)	

No. of subjects reporting at least one AEs post-treatment		0	1 (16.7)	2 (33.3)	1 (16.7)	2 (33.3)	1 (16.7)	7 (19.4)	1 (8.3)	3 (25.0)	0	0	0	1 (11.1)	1 (14.3)
Metabolism and Nutrition	Decreased Appetite	0	0	0	0	1 (16.7)	1 (16.7)	2 (5.6)	1 (8.3)	0	0	0	0	0	0
Musculoskeletal and Connective Tissue Disorders	Back Pain	0	0	0	0	0	1 (16.7)	1 (2.8)	1 (8.3)	1 (8.3)	0	0	0	0	0
	Myalgia	0	0	1 (16.7)	0	0	0	1 (2.8)	0	0	0	0	0	0	0
	Pain in Extremity	0	0	0	0	1 (16.7)	1 (16.7)	2 (5.6)	0	0	0	0	0	0	0
Nervous System Disorders	Dizziness Postural	0	1 (16.7)	0	1 (16.7)	0	0	2 (5.6)	0	0	0	0	0	0	0
	Headache	0	0	1 (16.7)	0	1 (16.7)	1 (16.7)	3 (8.3)	0	1 (8.3)	0	0	0	0	0
General disorders and administration site conditions	Pyrexia	0	0	0	0	0	0	0	0	0	0	0	0	1 (11.1)	0
Respiratory, thoracic, and mediastinal disorders	Cough	0	0	0	0	0	0	0	0	0	0	0	0	0	1 (14.3)

N=Number of subjects with non-missing values; n/% = Number/percentage of subjects with the given characteristics.

a Cohort 1 received 2 dose levels, 5 and 100 mg, separated by a washout period of 7 days.

b Cohort 2 received 2 dose levels, 25 and 200 mg, separated by a washout period of 7 days.

c Cohort 3 received 2 dose levels, 50 and 400 mg, separated by a washout period of 7 days.

Abbreviation: SAD, single ascending dose; MAD, multiple ascending doses.

#### Single Ascending Dose Study

The most commonly occurring AE was headache in both the elderly and adult cohorts and was experienced by 2 (8.3%) and 3 (8.3%) subjects, respectively, and was considered to be possibly related. Decreased appetite, pain in extremity, and postural dizziness were observed in 2 (5.6%) subjects, each respectively; back pain and myalgia were experienced by 1 (2.8%) subject each, respectively. At least one AE was experienced by 7 (19.4%) and 1 (8.3%) subjects receiving TRC150094 and placebo, respectively. Decreased appetite and myalgia were considered as possibly related to the TRC150094. There were no AEs reported for the 5 mg dose. Two AEs were reported for the 50 and 200 mg doses. One subject each reported AEs for doses of 25, 100, and 400 mg in the adult cohort. As compared to one AE of dizziness in fasting state, there were no AEs in the fed state. AEs were mild in severity, except back pain (moderate severity was experienced by one subject taking dose 400 mg and pain in the extremity in one subject each taking 200 and 400 mg doses).

In the elderly cohort, 3 (12.5%) subjects on TRC150094 experienced AEs with 50 mg dose, while none was observed with 150 mg dose. In subjects receiving TRC150094, headache was experienced by 2 (8.3%) subjects and was considered as possibly related. There were no AE/SAEs reported in Part B (food effect) of the study. Fecal occult blood test was negative in all subjects, and renal biomarker, alpha-GST, was below limit of detection in all subjects. No clinically significant abnormalities were found in hematological and biochemical laboratory parameters during the course of the study. In addition, there were no abnormal clinically significant findings in 12-lead ECG (including QTc interval) (Supplementary Table S1) and vitals (heart rate and blood pressure) during the course of the study.

##### Multiple Ascending Dose Study

Two AEs were experienced by two subjects (one each in the placebo and test group, respectively) in the MAD study. Pyrexia and cough were experienced by one subject each (11.1 and 14.3%) receiving TRC150094 150 mg and placebo, respectively. Both AEs were mild and were unlikely related to the study drug. No clinically relevant differences were observed for ECG (including QTc interval) (Supplementary Table S2), vitals, and laboratory parameters, including renal biomarker from baseline to end of study.

### Plasma Pharmacokinetics Properties of TRC150094

#### Single Ascending Dose Study

**FIGURE 2:**
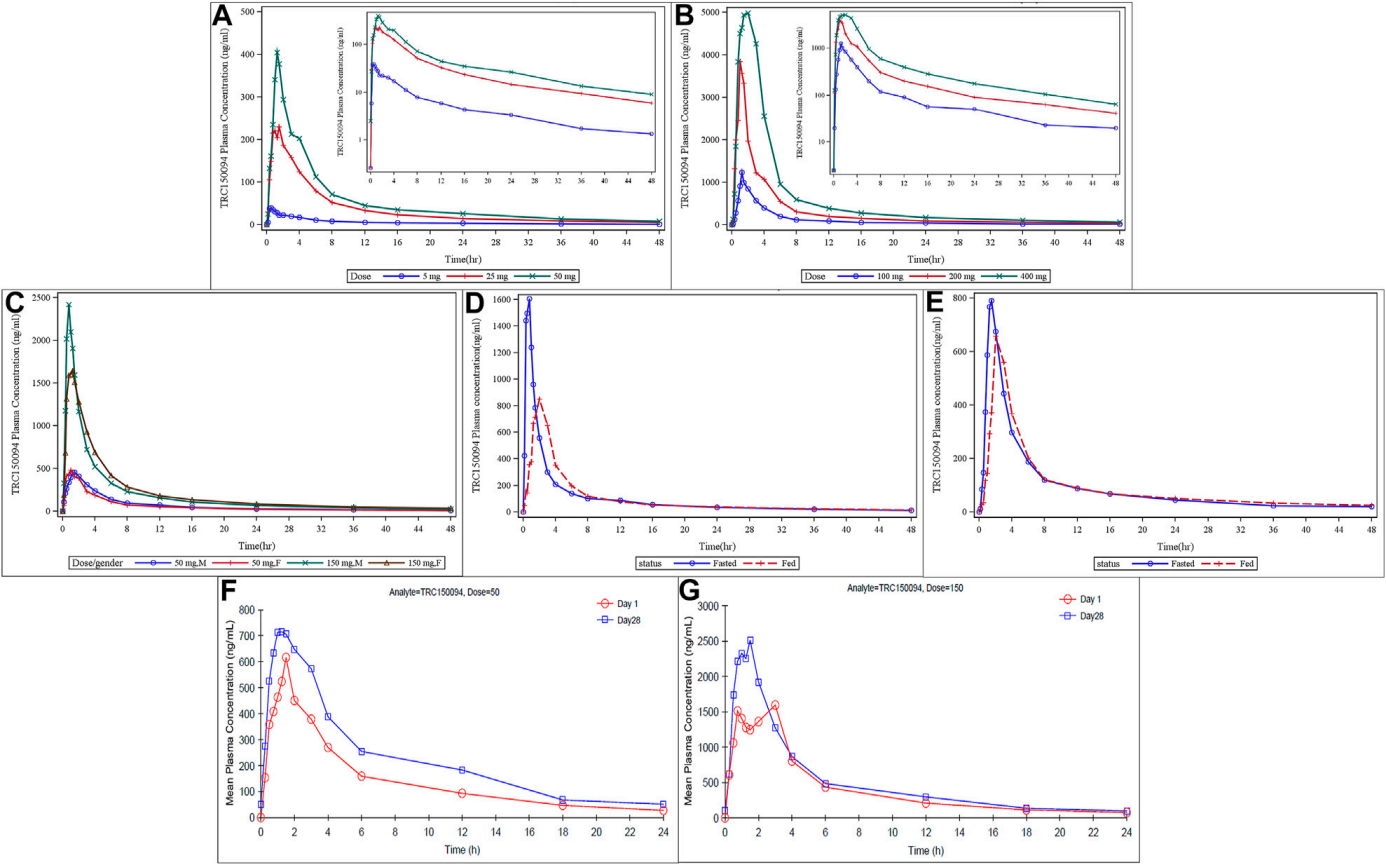
Mean plasma concentration of TRC150094 in single ascending dose study (adults, elderly subjects, and food effect study) and multiple ascending dose study. **(A,B)** In adults at lower doses (5, 25, and 50 mg) and at higher doses (100, 200, and 400 mg) **(C)** In elderly by dose and sex **(D,E)** Food status in females and in males **(F,G)** Mean plasma concentration of TRC150094 versus time curves after administration of TRC150094 on days 1 and 28 at 24 h, with 50 and 150 mg doses.

Plasma C_max_ and AUC increased dose-proportionally across the full dose range tested from dose level 5–400 mg (Figures 2A,B). TRC150094 was rapidly absorbed after oral administration as T_max_ was achieved within 2 h in most of the dose groups. The PK parameters, listed in Table 4, were similar in the elderly population (Figure 2C). No sex-related differences with C_max_, AUC_0-∞_, and T_max_ were observed. The dose proportionality was evaluated for elderly and adult subjects. The slope and its 90% CI for log C_max_ and log AUC0-∞ against log (dose) was 1.20 (1.13–1.28) and 1.03 (0.99–1.08), respectively. Meanwhile, the slope and its 95% CI for log C_max_ and log AUC_0-∞_ against log (dose) was 1.20 (1.11–1.30) and 1.03 (0.98–1.09), respectively. The AUC_0-∞_ was found to be dose-proportional across the full dose range tested in SAD (5–400 mg). The C_max_ and AUC values observed with 150 mg dose were proportionately higher (∼3 times) in the elderly cohort than that with the 50 mg dose. The AUC_0-∞_ was dose-proportional, and C_max_ was slightly more than proportional in the range of 50–150 mg.

**TABLE 4:** Summary of PK parameters of single dose study (SAD).

Variable (unit)		SAD (adult)						SAD (elderly)		SAD (food effect)	
	Dose (mg) N (M/F)	5^a^ 6 (4/2)	25^b^ 6 (5/1)	50^c^ 6 (3/3)	100^a^ 6 (4/2)	200^b^ 6 (5/1)	400^c^ 6 (3/3)	50 12 (7/5)	150 12 (7/5)	Fasted, 100 6 (3/3)	Fed, 100 6 (3/3)
**C**_**max**_ **(ng/ml)**	Mean ± SD	55.36 ± 22.65	266.489 ± 125.21	491.17 ± 172.51	1,602.98 ± 913.84	4,708.14 ± 2,960.82	7,153.41 ± 2052.63	557.06 ± 154.42	2,299.11 ± 868.25	1,619.58 ± 794.82	770.43 ± 129.05
	GM (95%CI)	50.67 (31.62, 79.15)	245.20 (135.10, 397.90)	467.22 (310.10, 672.20)	1,413.29 (644.10, 2,562.00)	3,928.92 (1,601.00, 7,815.00)	6,884.85 (4,999.00, 9,308.00)	537.41 (458.90, 655.20)	2,166.79 (1748.00, 2,851.00)	1,483.00 (785.30, 2,454.00)	761.20 (635.00, 905.90)
**AUC 0–24 (h·ng/ml)**	Mean ± SD	219.00 ± 58.50	1,434.47 ± 465.24	2,124.69 ± 485.22	4,590.58 ± 1,191.41	12,896.34 ± 5,806.48	25,108.27 ± 4,945.19	2,671.15 ± 654.60	8,405.46 ± 2,135.12	3,976.99 ± 394.03	3,929.60 ± 653.63
	GM (95%CI)	212.58 (157.80, 280.60)	1,379.28 (948.10, 1923.35)	2078.61 (1,616.00, 2,634.00)	4,470.21 (3,352.00, 5,839.00)	11,932.98 (6,837.00, 19,011.00)	24,684.91 (19,918.00, 30,298.00)	2,597.77 (2,257.00, 3,087.00)	8,167.36 (7,049.00, 9,762.00)	4,064.00 (3,587.00, 4,590.00)	3,789.00 (3,227.00, 4,431.00)
**AUC 0–48 (h·ng/ml)**	Mean ± SD	267.92 ± 83.15	1,673.31 ± 518.50	2,491.36 ± 661.03	5,281.86 ± 1,383.74	14,436.62 ± 6,422.69	27,814.48 ± 5,621.42	3,104.88 ± 707.61	9,576.71 ± 2,279.08	4,677.88 ± 580.03	4,526.18 ± 782.75
	GM (95%CI)	257.46 (180.90, 355.10)	1,613.98 (1,131.75, 2,217.08)	2,419.32 (1798.00, 3,185.00)	5,147.24 (3,841.00, 6,733.00)	13,365.46 (7,732.00, 21,196.00)	27,320.52 (21,915.00, 33,713.00)	3,029.85 (2,657.00, 3,554.00)	9,335.31 8,129.00, 11,025.00	4,656.00 (4,070.00, 5,307.00)	4,501.00 (3,740.00, 5,384.00)
**AUC 0-∞ (h·ng/ml)**	Mean ± SD	300.66 ± 101.79	1834.28 ± 542.31	2,744.43 ± 847.92	5,756.48 ± 1,467.45	15,436.31 ± 6,666.12	29,305.01 ± 6,377.20	3,415.58 ± 678.57	10,218.60 ± 2,389.89	5,119.05 ± 766.96	5,141.52 ± 1,079.02
	GM (95%CI)	287.20 (193.90, 407.40)	1772.81 (1,267.68, 2,403.95)	2,640.27 (1855.00, 3,634.00)	5,622.68 (4,225.00, 7,298.00)	14,338.23 (8,478.00, 22,450.00)	28,695.55 (22,613.00, 35,998.00)	3,351.98 (2,986.00, 3,846.00)	9,964.42 (8,700.00, 11,737.00)	5,077.00 (4,318.00, 5,942.00)	5,040.00 (4,008.00, 6,274.00)
**K**_**el**_ **(1/h)**	Mean ± SD	0.04 ± 0.01	0.04 ± 0.01	0.05 ± 0.01	0.05 ± 0.01	0.04 ± 0.01	0.05 ± 0.02	0.04 ± 0.01	0.05 ± 0.01	0.04 ± 0.01	0.04 ± 0.01
	GM (95%CI)	0.04 (0.03,0.05)	0.04 (0.03,0.05)	0.05 (0.03,0.06)	0.05 (0.03,0.06)	0.04 (0.03,0.05)	0.0482 (0.03,0.07)	0.04 (0.03,0.05)	0.0466 (0.03,0.04)	0.04 (0.03,0.06)	0.04 (0.03,0.05)
**T**_**1/2**_ **(h)**	Mean ± SD	16.64 ± 3.13	18.67 ± 3.99	15.63 ± 4.27	16.08 ± 5.75	17.43 ± 4.20	14.85 ± 3.95	19.10 ± 8.02	15.19 ± 3.21	17.46 ± 4.40	18.71 ± 4.07
	GM (95%CI)	16.40 (13.37, 19.92)	18.31 (14.48, 22.86)	15.16 (11.14, 20.11	15.40 (10.05, 22.11	17.01 (13.02, 21.83)	14.37 (10.71, 19.00)	17.84 (14.01, 24.19)	14.88 (13.15, 17.23	16.91 (12.84, 22.08	18.36 (14.43, 22.98)
**Tmax (h)**	Median (range)	0.80 (0.33–3.00)	1.01 (0.75–−3.00)	1.12 (0.33–−4.00)	1.37 (0.75–−3.00)	1.40 (0.50–−4.00)	1.75 (1.00–−3.00)	1.12 (0.50–−3.00)	0.87 (0.50–−2.00)	0.94 (0.33–−2.02)	2.01 (2.00–−3.00)

*N* = Number of subjects with non-missing values.

a Cohort 1 received 2 dose levels, 5 and 100 mg, separated by a washout period of 7 days.

b Cohort 2 received 2 dose levels, 25 and 200 mg, separated by a washout period of 7 days.

c Cohort 3 received 2 dose levels, 50 and 400 mg, separated by a washout period of 7 days.

Abbreviation: AUC, Area under curve; K_el_, elimination rate constant; SAD, single ascending dose; SD, standard deviation; T_max_, time to reach C_max_.

Compared to the fasting state, the C_max_ of TRC150094 in the fed state was reduced by approximately 50% in the food effect study. However, there was no effect of food on AUC (Figures 2D,E).

The amount of drug recovered in urine at the end of the collection period (48 h) was approximately 20% of the dose administered (100 mg), and more than 80% of this amount was recovered in 24 h. The renal clearance was approximately 4.22 L/h and 2.07 L/h in adults and the elderly, respectively.

#### Multiple Ascending Dose Study

TRC150094 was absorbed with the T_max_ similar to single-dose and eliminated with a median half-life of approximately 6–7 h (Table 5). There was no significant difference in T_max_ between Day 1 and Day 28 at each dose level [with a *p* value of 0.744 (50 mg) and 0.125 (150 mg)]. TRC150094 showed approximately 30% accumulation while achieving a steady-state within a week (Figures 2F,G).

**TABLE 5:** Summary of PK parameters of multiple dose study (MAD).

Variable (unit)		50 mg (*N* = 12)		150 mg (*N* = 9)	
		Day 1	Day 28	Day 1	Day 28
Tmax (h)*	Median (Range)	1.38 (0.25–4.00)	1.13 (0.25–3.00)	1.50 (0.50–3.00)	1.00 (0.75–1.50)
**C**_**max**_ **(ng/ml)**	Mean ± SD	869.32 ± 350.29	1,048.58 ± 381.02	2,583.06 ± 790.49	3,311.59 ± 699.20
	GM (95%CI)	800.27 (646.76, 1,091.88)	992.82 (806.50, 1,290.66)	2,462.57 (1975.44, 3,190.68)	3,244.59 (2,774.14, 3,849.04)
**AUClast (ng.h/ml)**	Mean ± SD	3,388.92 ± 884.45^a^	6,288.65 ± 1955.92	9,644.49 ± 2,730.91^a^	14,217.39 ± 2,524.40
	GM (95%CI)	3,292.26 (2,826.98, 3,950.87)	5,996.61 (5,045.94, 7,531.37)	9,319.60 (7,545.33, 11,743.7)	14,034.9 (12,277.0, 16,157.8)
**AUC0-inf (ng.h/ml)**	Mean ± SD	3,683.53 ± 936.03^a^	6,463.85 ± 1961.86	10,413.09 ± 3,037.68^a^	14,410.00 ± 2,568.95
	GM (95%CI)	3,582.61 (3,088.82, 4,278.25)	6,178.82 (5,217.36, 7,710.34)	10,047.6 (8,078.13, 12,748.1)	14,223.90 (12,435.30, 16,384.70)
**AUC 0–24 (h·ng/ml)**	Mean ± SD	3,388.92 ± 884.45	5,307.37 ± 1,474.97	9,644.49 ± 2,730.91	12,088.55 ± 2,265.08
	GM (95%CI)	3,292.26 (2,826.98, 3,950.87)	5,105.96 (4,370.24, 6,244.51)	9,319.60 (7,545.33, 11,743.7)	11,915.6 (10,347.5, 13,829.6)
**AUC 0–48 (h·ng/ml)**	Mean ± SD	-	6,089.80 ± 1727.59	-	13,594.22 ± 2,406.65
	GM (95%CI)	-	5,853.08 (4,992.16, 7,187.44)	-	13,420.8 (11,744.3, 15,444.1)
**AUC 0–96 (h·ng/ml)**	Mean ± SD	-	6,382.18 ± 1901.64	-	14,249.44 ± 2,502.39
	GM (95%CI)	-	6,110.85 (5,173.95, 7,590.42)		14,070.6 (12,325.9, 16,172.9)
**Kel (1/h) (0–24 h)**	Mean ± SD	0.10 ± 0.02	0.11 ± 0.01	0.10 ± 0.02	0.11 ± 0.02
	GM (90%CI)	0.10 (0.09, 0.12)	0.11 (0.10, 0.12)	0.10 (0.09, 0.11)	0.10 (0.09, 0.12)
**Kel (1/h) (0–96 h)**	Mean ± SD	-	0.06 ± 0.02	-	0.04 ± 0.01
	GM (95%CI)	-	0.05 (0.04, 0.07)	-	0.04 (0.03, 0.05)
T1/2 (h) (0–96 h)*	Mean ± SD	-	14.063 ± 4.49	-	17.629 ± 4.13
	GM (95%CI)	-	13.31 (11.05, 17.08)	-	17.23 (14.46, 20.80)
**Accumulation Ratio [AUC0-12]**	Mean ± SD	1.54 ± 0.27		1.27 ± 0.17	
	GM (95%CI)	1.57 (1.42, 1.75)		1.27 (1.14, 1.41)	
**Accumulation Ratio [AUC0-24]**	Mean ± SD	1.57 ± 0.27		1.29 ± 0.18	
	GM (95%CI)	1.60 (1.46, 1.77)		1.28 (1.15, 1.43)	

a For day 1, AUClast and AUC0-inf from 0 to 24 plasma concentration-time profile.

GM: Geometric mean. 95% CI: 95% confidence interval.

TRC150094 showed a dose-proportional increase in the rate of absorption (C_max_) and the extent of absorption (AUC_0-24_) upon a single dose. On Day 28, the t_1/2_ was 13.61 and 16.4 h for 50 and 150 mg doses, respectively (Table 5).

The mean accumulation ratio between Day 1 and Day 28 as a measure of AUC_0-12_ and AUC_0-24_ were 1.54 and 1.57 at 50 mg dose level and 1.27 and 1.29 at 150 mg dose levels, respectively (Table 5). Hence, TRC150094 tends to accumulate upon multiple-dose administration.

Upon administration of single and multiple oral doses of TRC150094 150 mg, the parent compound was rapidly and extensively converted to its metabolites. Refer to the structure of the TRC150094 and its metabolites is shown in Supplementary Figure S1. There was no significant difference observed in T_max_ between Day 1 and Day 28 at the 150 mg dose level. The metabolites also followed the parent in terms of T_max_ and t_1/2_. The mean C_max_ and AUC_0-24_ of metabolites confirm that M-1 was the major circulating metabolite. All metabolites showed an accumulation of less than 20%, while steady-state was reached after 2–3 doses (Supplementary Table S3). Concentration-time profile for all the three metabolites of P15 is shown in the Supplementary Table S4 (plasma) and Supplementary Table S5 (urine).

### Pharmacodynamics Effect of TRC150094

Table 6 presents the comparison of early PD markers of TRC150094 50 and 150 mg doses with placebo after adjustment of change in the body weight from baseline.

**TABLE 6:** Comparison of TRC150094 50 and 150 mg with placebo for percentage change of pharmacodynamic markers using Dunnett’s test while adjusting for the change in body weight.

Comparison	Apo B	Triglycerides	MATSUDA index	HOMA-IR	Hepatic fat by MRS
	Difference between means 95% CI (LL-UL), *p* value	Difference between means 95% CI (LL-UL), *p* value	Difference between means 95% CI (LL-UL), *p* value	Difference between means 95% CI (LL-UL), *p* value	Difference between means 95% CI (LL-UL), *p* value
TRC150094 50 mg–Placebo	−22.49^a^ (−40.12, -4.85), 0.01	−43.01 (−89.54, 3.51), 0.07	−17.66 (−163.45, 128.12), 0.93	−89.00 (−324.63, 146.62), 0.56	−4.57 (−26.58, 17.44), 0.84
TRC150094 150 mg–Placebo	−12.33 (−31.02, 6.35), 0.21	−19.83 (−69.13, 29.47), 0.49	94.34 (−60.14, 248.81), 0.24	−29.19 (278.87–220.48), 0.83	−0.27 (−23.59, 23.06), 0.99

a Comparisons significant at the 0.05 level. 95% CI (LL-UL):95% Confidence Interval (CI) (Lower limit, Upper limit).

Abbreviation: ApoB, apolipoprotein B, HOMA-IR, Homeostatic model assessment-insulin resistance; MRS, magnetic resonance spectroscopy.

#### Evaluation of Pharmacodynamics Markers in the Multiple Ascending Dose Study

For ApoB, there was a significant reduction from baseline observed at the lower dose of TRC150094 50 mg (−2.34%) vs. placebo (13.24%) (*p* = 0.01), whereas TRC150094 150 mg (8.78%) (*p* = 0.12) exhibited no significant difference. A trend towards reduction was observed between baseline and end study triglycerides values for TRC150094 50 mg vs. placebo (44 vs. 24%, respectively; *p* = 0.08). However, triglyceride at 150 mg dose (*p* = 0.42) showed no clinically significant reduction. For MATSUDA Index, HOMA-IR, Hepatic Fat by MRS, triglycerides, OUES, and Peak V_O2_, there was no significant difference in % change observed for TRC150094 50 and 150 mg vs. placebo. Furthermore, the difference observed between baseline and end study values of Hepatic Fat by MRS were, for TRC150094 50 mg (31%) and 150 mg (27%) and placebo (25%).

## Discussion

TRC150094, a novel mitochondrial modulator, was evaluated primarily for its safety and PK properties in this study. The safety, tolerability, and PK profile of single and multiple ascending doses of TRC150094 (5–400 mg) administered to healthy overweight/obese subjects was studied for up to 28 days.

Analysis of safety data showed that TRC150094 was well-tolerated when administered in fed or fasted conditions. All the AEs reported were mild to moderate in nature, and none was considered definitely or probably related to treatment. The most frequently occurring AE, considered to be possibly related to the treatment, was headache in both the elderly and adult cohorts. Most of the AEs resolved on their own without the need for rescue medications. There were no abnormal clinical or laboratory findings for cardiac or renal parameters. Additionally, there were no SAEs or withdrawals due to AEs in both studies.

The PK data demonstrated that oral doses of TRC150094 were rapidly absorbed. The T_max_ ranged from 0.25 to 4 h, and the elimination half-life ranged from 15 to 18 h, making it suitable for once-daily dosing. The half-life is similar to commonly used antidiabetic drugs with once-daily dosing recommendations with comparable terminal half-life, such as canagliflizin (10.6–13.1 h), Dapagliflozin (12.9 h), empagliflozin (11.7–19 h), and sitagliptin (8–14 h). The PK of TRC150094 was similar irrespective of age and sex. The consumption of food delayed the absorption of TRC150094; however, the extent of absorption remained unchanged. Therefore, food does not interfere with the overall absorption of the drug. The metabolites of TRC150094 are glucuronide and sulfate conjugates, and 20% of the drug is excreted unchanged in the urine. TRC150094 is primarily metabolized by the Phase-II conjugation reactions, which are not catalyzed by the cytochrome P450 system. As evaluated in isolated human hepatocytes (non-clinical study), TRC150094 does not have a positive inductive effect on CYP3A and CYP1A isozymes and thus have a lesser likelihood of CYP-mediated drug-drug interactions.

The American College of Cardiology/American Heart Association guidelines acknowledge that ApoB and non-HDL-C are more accurate markers of cardiovascular risk than LDL-C (Grundy et al., 2019; Fonseca et al., 2020). Exploratory analysis for PD markers in overweight and obese individuals demonstrated an improving trend in HOMA-IR, MATSUDA Index, and reduction in triglycerides and hepatic fat. OUES and peak V_O2_, however, did not show a change. A significant reduction was noted in ApoB levels at 50 mg compared to placebo, but not at 150 mg. Overall, TRC150094 50 mg dose showed a relatively better effect than the 150 mg dose. There was a variable response on glycaemic and lipid parameters at a higher dose of 150 mg. This might suggest an upregulation of lipolytic and gluconeogenetic pathways beyond a threshold, at the higher dose. Nonetheless, these findings show trends similar to the studies on Wistar rats and obese ZSF rats, showing prevention of accumulation of visceral fat, which was associated with enhanced mitochondrial respiratory chain activity and fatty acid oxidation (Cioffi et al., 2010; Zambad et al., 2011).

A limitation of our study is the relatively small sample size, which needs to be considered when interpreting the PD markers. The preclinical models, which showed improvement in PD parameters, had overt diabetes associated with marked obesity, dyslipidemia, and hypertension, and the duration of treatment was 24 weeks. The subjects in this study had baselines that were mildly deranged at best, and the duration of treatment was limited to 28 days. These observations are similar to those reported in an exploratory study by Fleur van der Valk et al., wherein TRC150094 was administered for 28 days to obese insulin-resistant males (van der Valk et al., 2014). In a subset with TG > 1.64 mmol/L, serum TG and intrahepatic TG exhibited a decreasing trend, although this did not translate into improvement in insulin sensitivity. It is possible that the treatment of 4 weeks was not of sufficient duration for improving insulin resistance. Based on these results, TRC150094 was subsequently evaluated in phase 2 study in diabetic patients with hypertension and dyslipidemia at doses ranging from 25 to 75 mg administered for 24 weeks (Clinical Trial Registry of India registration number: CTRI/2013/03/003444 and CTRI/2013/03/003445, manuscript under review). Based on the phase 2 study, a multinational phase 3 study is ongoing to determine the benefit of 45 mg dose on the concurrent improvement of all the risk factors for cardiovascular disease, i.e., HbA1c, MAP, and non-HDL cholesterol (ClinicalTrials.gov registry number: NCT03254446).

## Conclusion

Overall, TRC150094 has shown a promising safety profile in line with preclinical studies. The current Phase-I SAD and MAD studies reconfirm the safety of the investigational product. TRC150094 is a mitochondrial modulator that restored metabolic flexibility and improved insulin resistance in preclinical models. Translation of the benefit observed in animal models to the clinical situation needs to be evaluated further in those with distinctly deranged glycemic and lipid profiles, i.e., CMBCD with concurrent diabetes, dyslipidemia, and hypertension.

## Data Availability

The raw data supporting the conclusions of this article will be made available by the authors, without undue reservation.
