# Supplementary material for: Safety, Tolerability, and Pharmacokinetics of a Novel Mitochondrial Modulator, TRC150094, in Overweight and Obese Subjects: A Randomized Phase-I Clinical Trial
Source: Front Pharmacol. 2021 Sep 17;12:729424. doi: 10.3389/fphar.2021.729424 (PMC8485898; doi:10.3389/fphar.2021.729424)
Supplement: Supplementary file 1 [file DataSheet1.docx]

Supplementary Material

# Supplementary Data

## Analytical method:

Instrumentation: API 4000 (Applied Biosystems, MDS Sciex, USA) and TSQ VANTAGE (Thermo Fisher Scientific, USA) triple stage quadruple mass spectrometer connected with LC20-AD solvent delivery module, CTO -20A column oven and SIL-HTc auto-sampler (Shimadzu, Japan) were used for analysis of TRC150094 and its metabolites in plasma and urine.

Analysis Conditions: Isocratic, reverse phase liquid chromatography with electrospray ionization in positive mode used for analysis of parent and metabolites in plasma and urine samples after solid phase extraction. During quantitation, the calibration lines were constructed by weighted (1/*x^2^*) least-square linear regression analysis of the peak area ratio of analytes to IS versus the concentrations.

Liquid chromatography-mass spectrometry parameters and linearity range of methods presented below

| **Study (Code)** | **Analytes** | **Matrix** | **HPLC parameters** | **Triple Quadrupole**  **Mass parameters** | **Linearity Range** |
| --- | --- | --- | --- | --- | --- |
| **SAD**  **(CT/P015/MS/09/01_01)** | TRC150094 | Plasma | Shimadzu HPLC, Beta basic C8 150* 4.6mm, 5µ column  Mobile Phase: Methanol: 5 mM Ammonium Acetate: (70:30) %v/v, isocratic, 0.7 mL/min. flow rate.  Injection Volume: 2 µL  Column oven temperature: 40°C | API 4000  ESI Positive ion-mode MRM Transitions (Q1/Q3, m/z): TRC150094: 329.30/161.30,329.30/169.30 Internal Standard (Atorvastatin):559.30/440.40,559.30/466.20 Dwell Time-100ms | 5.000 ng/mL to 2000.000 ng/mL |
| **SAD**  **(CT/P015/MS/09/01_01)** | TRC150094 | Plasma | Shimadzu HPLC, Beta basic C8 150* 4.6mm, 5µ column  Mobile Phase: Methanol : 5 mM Ammonium Acetate : (70:30) %v/v, isocratic, 0.7 mL/min. flow rate  Injection Volume: 5 µL  Column oven temperature: 40°C | API 4000  ESI Positive ion-mode MRM Transitions (Q1/Q3, m/z): TRC150094: 329.30/161.30,329.30/169.30 Internal Standard (Atorvastatin):559.30/440.40,559.30/466.20 Dwell Time-100ms | 0.500 ng/mL to 200.000 ng/mL |
| **SAD**  **(CT/P015/MS/09/01_01)&**  **MAD (CT/P015/MS/09/01_02)** | TRC150094 | Urine | Shimadzu HPLC, ZORBAX SB-C18 75*4.6mm, 3.5µ column.  Mobile Phase:  Methanol: 5 mM Ammonium Acetate: (50:50) %v/v , isocratic, 0.5 mL/min. flow rate. Injection Volume: 2 µL  Column oven temperature: 40°C | API 4000  ESI Positive ion-mode MRM Transitions (Q1/Q3, m/z): TRC150094: 329.30/161.30,329.30/257.20 Internal Standard (TRC041195):216.20/137.20 Dwell Time-100ms | 0.100 µg/mL to 40.000 µg/mL |
| **MAD (CT/P015/MS/09/01_02)** | TRC150094 | Plasma | Shimadzu HPLC, ZORBAX SB-C18 75*4.6mm, 3.5µ column  Mobile Phase: Methanol : 5 mM Ammonium Acetate : (50:50) %v/v , isocratic, 0.5 mL/min. flow  Injection Volume: 2 µL  Column oven temperature: 40°C | API 4000  ESI Positive ion-mode MRM Transitions (Q1/Q3, m/z): TRC150094: 329.30/161.30,329.30/257.20 Internal Standard (TRC041195):216.20/137.20 Dwell Time-100ms | 5.000 ng/mL to 2000.000 ng/mL |
| **MAD (CT/P015/MS/09/01_02)** | Metabolites  ( M1,M2 & M3) | Plasma | Shimadzu HPLC, ZORBAX XDB-C18 100*4.6mm, 3.5µ column  Mobile Phase: Methanol : 5 mM Ammonium Acetate :Acetonitrile (45:45:10) %v/v/v, isocratic, 0.5 mL/min. flow  Injection Volume: 8 µL  Column oven temperature: 40°C | API 4000  ESI Positive ion-mode MRM Transitions (Q1/Q3, m/z): Metabolite M1: 505.30/329.30  Metabolite M2: 505.30/329.30,505.30/311.40  Metabolite M3: 409.30/329.40 Internal Standard (TRC041195):216.20/137.20 Dwell Time-100ms  TSQ VANTAGE  ESI Positive ion-mode MRM Transitions (Q1/Q3, m/z): Metabolite M1: 505.166/161.243,505.166/257.334  Metabolite M2: 505.169/161.116,505.169/257.275Metabolite M3: 409.148/161.157, 409.148/257.192, 409.148/329.280 Internal Standard (TRC041195):216.083/79.079, 216.083/136.066  Dwell Time-200ms | Metabolite1-5.000 ng/mL to 2000.000 ng/mL    Metabolite2-5.000 ng/mL to 1000.000 ng/mL    Metabolite3-1.000 ng/mL to 500.000 ng/mL |
| **MAD (CT/P015/MS/09/01_02)** | Metabolites  ( M2 & M3) | Urine | Shimadzu HPLC, ZORBAX XDB-C18 100*4.6mm, 3.5µ column  Mobile Phase: Methanol : 10 mM Ammonium Acetate :Acetonitrile (45:45:10) %v/v/v, isocratic, 0.5 mL/min. flow  Injection Volume: 10 µL  Column oven temperature: 40°C | TSQ VANTAGE  ESI Positive ion-mode MRM Transitions (Q1/Q3, m/z): Metabolite M2: 505.100/161.130,505.100/257.200Metabolite M3: 409.148/161.090, 409.148/257.200 Internal Standard (TRC150129):343.206/297.183,  Dwell Time-200ms | Metabolite2-  50.000 ng/mL to 5000.000 ng/mL  Metabolite3-10.000 ng/mL to 1000.000 ng/mL |
| **MAD (CT/P015/MS/09/01_02)** | Metabolite1  (hydrolysed to TRC150094) | Urine | Shimadzu HPLC, ZORBAX SB-C18 75*4.6mm, 3.5µ column  Mobile Phase: Methanol : 5 mM Ammonium Acetate : (50:50) %v/v , isocratic, 0.5 mL/min. flow  Injection Volume: 2 µL  Column oven temperature: 40°C | API 4000  ESI Positive ion-mode MRM Transitions (Q1/Q3, m/z): TRC150094: 329.30/161.30,329.30/257.20  Metabolite-1: 505.30/329.30 Internal Standard (TRC041195):216.20/137.20 Dwell Time-100ms | 0.100 µg/mL to 40.000 µg/mL |

Performance of the assay during the course of sample analysis: The quality control (QC) samples analysed were within acceptance criteria as defined in regulatory guidance for bioanalysis.

The intra-day assay performance was evaluated based on accuracy, within ±15% of respective nominal concentration, of bracketing quality control (batch QC) samples. A set of QCs included LQC, MQC and HQC were analysed before and after study sample analysis. The QCs were within the acceptance criteria of accuracy and minimum 4 out 6 (67.6%) QC satisfied accuracy criteria.

The inter-day precision and accuracy for TRC150094 plasma and urine sample bioanalysis were calculated considering all QC samples (global) analysed during the course of respective Phase 1 (SAD and MAD) study sample analysis. The precision was in the range of 3.41 to 9.43 % and accuracy in the range of 96.18 to 103.24 %. For metabolites during MAD sample analysis (plasma and urine), the global precision and accuracy was in the range of 5.25 to 11.78 % and 94.64 to 104.03%, respectively.

**Parent:**

| **Study** | **Matrix** | **Inter day QC** | |
| --- | --- | --- | --- |
|  |  | **Precision** | **Accuracy** |
| SAD | Plasma | 4.05 to 9.43 % | 96.75 to 103.27 % |
| SAD | Urine | 3.41 to 8.47 % | 97.15 to 102.33 % |
| MAD | Plasma | 4.47 to 6.21 % | 97.32 to 101.24 % |
| MAD | Urine | 5.01 to 7.69 % | 96.18 to 99.67 % |

**Metabolite:**

Metabolite 1:

| **Study** | **Matrix** | **Inter day QC** | |
| --- | --- | --- | --- |
|  |  | **Precision** | **Accuracy** |
| MAD | Plasma | 6.67 to 7.99 % | 98.52 to 101.11 % |
| MAD | Urine | 6.05 to 10.03 % | 99.13 to 104.03 % |

Metabolite 2:

| **Study** | **Matrix** | **Inter day QC** | |
| --- | --- | --- | --- |
|  |  | **Precision** | **Accuracy** |
| MAD | Plasma | 6.18 to 9.45 % | 99.30 to 99.64 %. |
| MAD | Urine | 7.35 to 10.07 % | 97.63 to 99.85 %. |

Metabolite 3:

| **Study** | **Matrix** | **Inter day QC** | |
| --- | --- | --- | --- |
|  |  | **Precision** | **Accuracy** |
| MAD | Plasma | 7.54 to 11.78 % | 97.03 to 100.98 %. |
| MAD | Urine | 5.25 to 7.48 % | 94.64 to 96.60%. |

# Supplementary Figures and Tables

# Supplementary Tables

**Supplementary Table S1: Change from baseline for QTc interval on Electrocardiogram (ECG) in the single ascending dose (SAD) study**

| **Mean change in QTc from baseline (msec)** | **Non-Elderly** | | | | | | | | **Elderly** | | | |
| --- | --- | --- | --- | --- | --- | --- | --- | --- | --- | --- | --- | --- |
|  | **TRC150094** | | | | | | **Total** | | **TRC150094** | | **Total** | |
|  | **5**  **mg** | **25**  **mg** | **50**  **mg** | **100**  **mg** | **200**  **mg** | **400**  **mg** | **TRC150094** | **Placebo** | **50**  **mg** | **150**  **mg** | **TRC150094** | **Placebo** |
| **0.5hr-predose** | -3.67 | -3.28 | 1.22 | 1.67 | -3.56 | -1.17 | -1.52 | -2.53 | -4.56 | -0.86 | -3.00 | -8.33 |
| **1hr-predose** | -5.11 | 4.72 | 3.78 | 0.06 | -2.50 | -3.94 | -0.50 | -8.97 | -0.17 | -1.89 | -1.32 | -7.33 |
| **4hr-predose** | -4.50 | 2.67 | 1.72 | 6.34 | 1.67 | 2.39 | 1.71 | 4.50 | -0.98 | -2.86 | -1.91 | -6.92 |
| **8hr-predose** | -4.16 | -0.11 | 4.39 | -5.44 | -2.44 | -1.17 | -1.49 | 8.39 | -0.78 | -3.81 | -2.29 | -7.00 |
| **12hr-predose** | -2.00 | 2.89 | 4.11 | 2.67 | 6.33 | 4.89 | 3.15 | 5.39 | 3.47 | -3.33 | -0.22 | -2.50 |
| **24hr-predose** | -7.05 | 2.33 | 0.00 | 0.95 | -1.83 | -4.22 | -1.64 | 2.50 | 0.42 | -6.83 | -3.21 | -6.08 |
| **48hr-predose** | -5.50 | -5.61 | 2.28 | 7.11 | -2.50 | -0.92 | -0.92 | 2.42 | -2.97 | -3.56 | -3.26 | -9.58 |

**Supplementary Table S2: Change from baseline for QTc interval on Electrocardiogram (ECG) in the multiple ascending dose (MAD) study**

| **Change from baseline (Mean)** | **Male** | | | **Female** | | |
| --- | --- | --- | --- | --- | --- | --- |
|  | **TRC150094** | | **Placebo n=5** | **TRC150094** | | **Placebo n=2** |
|  | **50mg n=9** | **150mg n=5** |  | **50mg n=3** | **150mg n=4** |  |
| 0.5hr – pre-dose day 1 | -4.04 | -6.93 | -0.07 | -1.89 | 2.42 | -4.17 |
| 1hr – pre-dose day 1 | -3.70 | -8.93 | 1.13 | 2.11 | 3.00 | 1.33 |
| 4hr – pre-dose day 1 | 7.15 | -1.93 | 10.67 | -3.89 | 15.50 | 3.33 |
| 8hr – pre-dose day 1 | 10.04 | -14.73 | 4.40 | 3.00 | 8.58 | 3.00 |
| 12hr – pre-dose day 1 | 15.30 | -3.40 | 5.33 | 5.11 | 16.67 | 4.50 |
| 2 days – pre-dose day 1 | 3.52 | -9.93 | 3.33 | 4.22 | 4.25 | 0.50 |
| Day28 – pre-dose day 1 | -11.52 | -6.33 | -2.47 | -13.67 | 0.92 | 0.33 |

**Supplementary Table S3: Plasma pharmacokinetic parameters of TRC150094 metabolites (Multiple Ascending Dose study)**

| **Variable (units)** |  | **Metabolite 1 (N=9)** | | **Metabolite 2 (N=9)** | | **Metabolite 3 (N=9)** | |
| --- | --- | --- | --- | --- | --- | --- | --- |
|  |  | **Day 1** | **Day 28** | **Day 1** | **Day 28** | **Day 1** | **Day 28** |
| Tmax (h)* | Median (Range) | 2.00 (0.75-3.00) | 1.00 (0.75-2.00) | 1.50 (0.25-3.00) | 1.00 (0.75-1.50) | 3.00 (1.25-4.00) | 2.00 (1.25-3.00) |
| C_max_ (ng/ml) | Mean ± SD | 3328.54 ± 912.01 | 5418.72 ± 1442.93 | 2251.14 ± 937.86 | 2436.01 ± 940.31 | 94.44 ± 28.79 | 116.42 ± 42.71 |
|  | GM (95%CI) | 3207.87  (2627.51, 4029.57) | 5264.12  (4309.58, 6527.85) | 2082.49  (1530.24, 2972.03) | 2279.15  (1713.22, 3158.80) | 90.04  (72.336, 116.60) | 109.95  (83.59, 149.25) |
| AUClast (ng.h/mL) | Mean ± SD | 15559.50 ± 2831.49^#^ | 22608.38 ± 7609.98 | 8402.39 ± 4236.88^#^ | 8695.35 ± 4113.43 | 657.52 ± 240.57^#^ | 905.45 ± 357.48 |
|  | GM (95%CI) | 15338.60  (13383.0, 17736.0) | 21592.30  (16758.8, 28457.9) | 7492.39  (5145.65, 11659.1) | 7900.06  (5533.50, 11857.2) | 622.35  (472.599, 842.43) | 845.65  (630.663, 1180.24) |
| AUC0-inf (ng.h/mL) | Mean ± SD | 16648.34 ± 3220.76^#^ | 22938.64 ± 7803.72 | 8955.08 ± 4583.23^#^ | 8921.37 ± 4130.28 | 715.78 ± 263.66^#^ | 947.55 ± 351.20 |
|  | GM (95%CI) | 16385.30  (14172.70, 9124.00) | 21886.00  (16940.20,28937.10) | 7980.69  (5432.10, 2478.10) | 8133.58  (5746.56, 2096.20) | 677.04  (513.11, 918.44) | 890.99  (677.60, 1217.51) |
|  | GM (95%CI) | 15338.60  (13383.0, 17736.0) | 17982.80  (14602.8, 22533.9) | 7492.39  (5145.65, 11659.1) | 7010.92  (4969.58, 10385.3) | 622.35  (472.60, 842.43) | 709.99  (526.72, 998.67) |
| AUC 0-48 (h·ng/ml) | Mean ± SD | - | 21260.41 ± 6535.19 | - | 8483.10 ± 3890.24 | - | 876.96 ± 336.37 |
|  | GM (95%CI) | - | 20450.80  (16237.00, 26283.80) | - | 7747.57  (5492.80, 11473.40) | - | 821.40  (618.40, 1135.51) |
| AUC0-96 (ng.h/mL) | Mean ± SD | - | 22625.23 ± 7591.60 | - | 8803.02 ± 4066.75 | - | 923.62 ± 353.05 |
|  | GM (95%CI) | - | 21616.20  (16789.80, 28460.60) | - | 8026.66  (5677.04, 11929.00) | - | 865.76  (652.24, 1195.00) |
| Kel  (1/h)  (0-24 hr) | Mean ± SD | 0.11 ± 0.02 | 0.09 ± 0.03 | 0.09 ± 0.03 | 0.10 ± 0.04 | 0.10 ± 0.02 | 0.11 ± 0.03 |
|  | GM (95%CI) | 0.11  (0.09, 0.13) | 0.09  (0.06, 0.12) | 0.09  (0.07, 0.12) | 0.10  (0.07, 0.14) | 0.09  (0.08, 0.11) | 0.11  (0.09, 0.13) |
| Kel  (1/hr)  (0-96 h) | Mean ± SD | - | 0.04 ± 0.01 | - | 0.05 ± 0.02 | - | 0.04 ± 0.01 |
|  | GM (95%CI) | - | 0.04  (0.03, 0.05) | - | 0.05  (0.04, 0.07) | - | 0.04  (0.03, 0.05) |
| T1/2 (h)  (0-96 h) | Mean ± SD | - | 18.54 ± 5.06 | - | 14.96 ± 5.21 | - | 17.57 ± 4.91 |
|  | GM (95%CI) | - | 17.91  (14.652, 22.42) | - | 14.08  (10.95, 18.96) | - | 16.99  (13.80, 21.35) |
| Accumulation Ratio [AUC0-24] | Mean ± SD | 1.19 ± 0.22 (0.93-1.63) | | 0.95 ± 0.14 (0.75-1.16) | | 1.18 ± 0.31 (0.68-1.68) | |
|  | GM (95%CI) | 1.17 (1.02, 1.36) | | 0.94 (0.84, 1.05) | | 1.14 (0.94, 1.41) | |
| # For day 1, AUClast and AUC0-inf from 0 to 24 plasma concentration time profile.  GM: Geometric mean  95% CI: 95% confidence interval | | | | | | | |

**Supplementary Table S4: Plasma concentration-time profile for Metabolite-1, Metabolite-2 and Metabolite-3 after administration of TRC150094 150mg**

| **Time (hr)** | **Metabolite 1 (n=9)**  **Mean (SD)** | | **Metabolite 2 (n=9)**  **Mean (SD)** | | **Metabolite 3 (n=9)**  **Mean (SD)** | |
| --- | --- | --- | --- | --- | --- | --- |
|  | **Day 1** | **Day 28** | **Day 1** | **Day 28** | **Day 1** | **Day 28** |
| **0** | 0.0 | 172.4 (81.3) | 0.0 | 52.8 (33.4) | 0.0 | 8.3 (2.4) |
| **0.25** | 273.2 (546.8) | 569.6 (789.8) | 687.8 (1171.1) | 415.2 (378.8) | 2.0 (4.0) | 11.0 (6.8) |
| **0.5** | 1122.3 (1219.6) | 1950.2 (1686.2) | 1050.5 (863.3) | 1112.0 (859.1) | 13.5 (17.4) | 25.2 (17.9) |
| **0.75** | 2004.4 (1623.5) | 3639.5 (2681.0) | 1352.3 (845.0) | 1676.1 (1066.2) | 32.4 (28.9) | 47.9 (33.2) |
| **1** | 2191.6 (1523.1) | 3986.5 (2132.1) | 1428.4 (901.6) | 1798.7 (784.3) | 47.3 (40.8) | 63.1 (32.5) |
| **1.25** | 2022.8 (1155.9) | 4153.9 (1695.8) | 1336.6 (966.2) | 1879.4 (1025.2) | 52.2 (39.8) | 76.2 (29.8) |
| **1.5** | 1987.7 (1036.9) | 4294.5 (1224.5) | 1278.5 (1010.1) | 1956.4 (894.9) | 55.4 (40.0) | 94.0 (31.2) |
| **2** | 2260.5 (999.6) | 3497.4 (945.0) | 1338.3 (838.9) | 1532.0 (860.1) | 63.4 (33.6) | 111.1 (36.6) |
| **3** | 2290.5 (761.8) | 2052.6 (576.9) | 1434.8 (847.1) | 918.3 (587.0) | 88.5 (31.1) | 102.8 (44.9) |
| **4** | 1528.8 (436.5) | 1291.6 (327.3) | 832.5 (541.3) | 520.1 (320.3) | 70.3 (25.5) | 74.9 (33.2) |
| **6** | 750.8 (207.8) | 686.0 (173.8) | 360.9 (231.5) | 253.5 (150.5) | 45.6 (18.7) | 45.2 (22.1) |
| **12** | 353.8 (117.4) | 349.1 (161.7) | 127.0 (77.7) | 125.1 (57.6) | 17.0 (8.9) | 18.7 (9.0) |
| **18** | 195.5 (34.7) | 232.5 (107.9) | 72.9 (40.8) | 74.1 (33.6) | 9.6 (4.8) | 9.8 (4.3) |
| **24** | 112.3 (38.6) | 171.7 (83.6) | 43.2 (28.3) | 53.6 (28.4) | 5.6 (2.5) | 6.9 (2.3) |
| **48** | - | 52.7 (41.7) |  | 13.6 (8.4) |  | 2.6 (1.1) |
| **72** | - | 25.3 (24.9) |  | 5.5 (6.1) |  | 0.6 (0.7) |
| **96** | - | 10.4 (8.1) |  | 2.1 (4.2) |  | 0.1 (0.3) |

**Supplementary Table S5: Urine concentration-time profile for Metabolite-1, Metabolite-2 and Metabolite-3 after administration of TRC150094 150mg.**

| **Time interval (hr)** | **Metabolite 1 (n=9)**  **Mean (SD)** | | **Metabolite 2 (n=9)**  **Mean (SD)** | | **Metabolite 3 (n=9)**  **Mean (SD)** | |
| --- | --- | --- | --- | --- | --- | --- |
|  | **Day 1** | **Day 28** | **Day 1** | **Day 28** | **Day 1** | **Day 28** |
| 0.0-4.0 | 98947.6 (72776.1) | 277736.0 (523906.3) | 11641.4  (9223.3) | 19871.1 (26937.8) | 349.1  (356.7) | 763.3  (1217.4) |
| 4.0-8.0 | 298926.9 (122292.5) | 234308.4 (197950.5) | 14394.0 (12918.3) | 7312.9  (5996.3) | 1176.6  (578.7) | 931.9  (512.5) |
| 8.0-12.0 | 73108.1 (34651.1) | 79868.4 (127673.4) | 3076.3  (1773.9) | 1887.8  (1579.7) | 531.3  (291.9) | 360.8  (397.9) |
| 12.0-24.0 | 27238.8 (11006.9) | 24505.9 (20724.5) | 1403.5  (771.6) | 972.2  (717.9) | 221.3  (113.3) | 118.4  (48.8) |
| 24.0-36.0 |  | 19543.3 (30440.5) |  | 555.9  (438.3) |  | 82.1  (68.3) |
| 36.0-48.0 |  | 16286.5 (32147.0) |  | 409.6  (354.1) |  | 74.9  (88.6) |

## Supplementary Figures


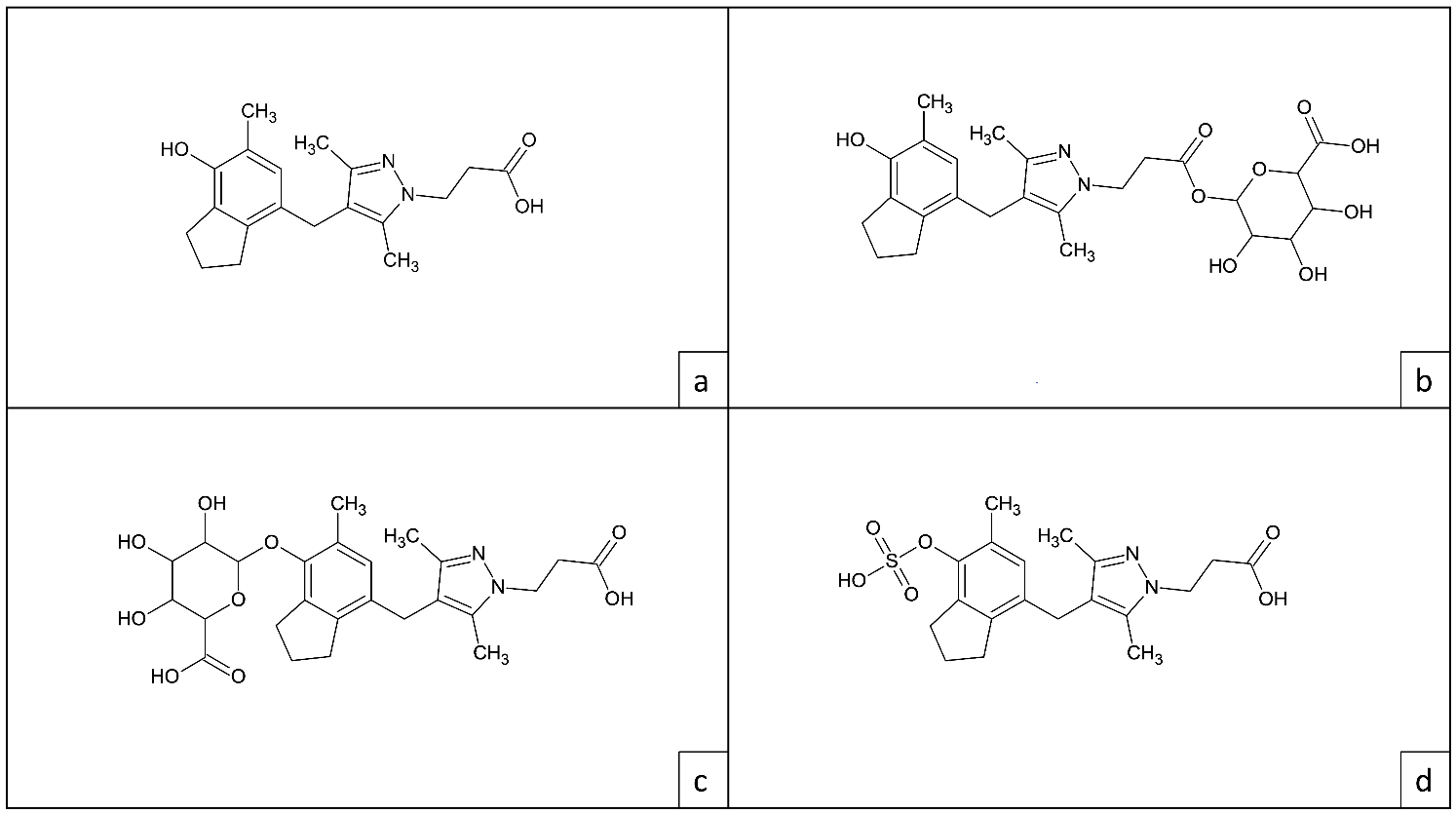


**Supplementary Figure S1.** The structure of the TRC150094 and its metabolites, a) TRC150094, b) TRC150231 (Metabolite M1), c) TRC150228 (Metabolite M2), d) TRC150227 (Metabolite M3).
